#  Comparative Bioinformatic Analysis of Active Site Structures in Evolutionarily Remote Homologues of α,β-Hydrolase Superfamily Enzymes 

**Published:** 2011

**Authors:** D.A. Suplatov, V.K. Arzhanik, V.K. Švedas

**Affiliations:** Faculty of Bioengineering and Bioinformatics, Lomonosov Moscow State University; Belozersky Institute of Physicochemical Biology, Lomonosov Moscow State University

**Keywords:** bioinformatics, comparative analysis, active site, structural alignment, α,β-hydrolases

## Abstract

Comparative bioinformatic analysis is the cornerstone of the study of enzymes’ structure-function relationship. However, numerous enzymes that derive from a common ancestor and have undergone substantial functional alterations during natural selection appear not to have a sequence similarity acceptable for a statistically reliable comparative analysis. At the same time, their active site structures, in general, can be conserved, while other parts may largely differ. Therefore, it sounds both plausible and appealing to implement a comparative analysis of the most functionally important structural elements – the active site structures; that is, the amino acid residues involved in substrate binding and the catalytic mechanism. A computer algorithm has been developed to create a library of enzyme active site structures based on the use of the PDB database, together with programs of structural analysis and identification of functionally important amino acid residues and cavities in the enzyme structure. The proposed methodology has been used to compare some α,β-hydrolase superfamily enzymes. The insight has revealed a high structural similarity of catalytic site areas, including the conservative organization of a catalytic triad and oxyanion hole residues, despite the wide functional diversity among the remote homologues compared. The methodology can be used to compare the structural organization of the catalytic and substrate binding sites of various classes of enzymes, as well as study enzymes’ evolution and to create of a databank of enzyme active site structures.

##  INTRODUCTION 

Comparative bioinformatic analysis is the cornerstone in the study of enzymes’ structure-function relationship. Multiple sequence comparisons have become a common tool in such an analysis. While a statistically significant sequence or tertiary structure similarity between proteins is justified as evidence of homology [1], some enzymes lose sequence similarity during natural selection and specialization from a common ancestor. Consequently, a bioinformatics analysis of remote homologues remains a bottleneck of existing methods for sequence comparison.

The protein’s structure is better conserved throughout evolution as compared to sequence [2, 3]. There are numerous examples of proteins that show sequence similarity close to random (roughly 8-15% identity considering gaps) but still adopt similar structures, contain identical or related amino acid residues in their active sites, and have similar catalytic mechanisms [4]. In contrast to commonly known sequence alignments [5-7], a three-dimensional alignment is based on the comparison of the geometric orientation of amino acid residues in tertiary structures, rather than on the biochemical properties of these residues at corresponding positions of primary structures [8]. Currently, there are almost 70,000 structures in the Protein Data Bank (PDB), and this number is constantly growing [9]. Accessibility of this information provides new opportunities for a comparative bioinformatic analysis. For example, the 3D-alignment of crystal structures has allowed to identify the relationship between distant members of Ntn-hydrolases family enzymes with low sequence similarity [10, 11]. It is therefore hoped that studying the structure-function relationship in enzyme families consisting of evolutionarily remote homologues using three-dimensional alignment could provide more significant clues as to a protein’s function, properties, and evolution than sequence alignment alone.

**Fig. 1:**
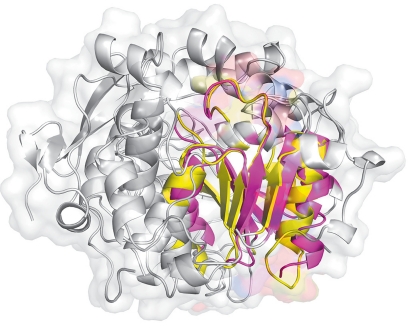
Structure alignment of lipase B from *C. antarctica* (1TCB) and hydroxynitrile lyase from *H. brasiliensis* (1YB6). Conserved parts containing the active site residues of two enzymes are marked in different colors.

The experience gained in a comparative analysis allows to assume that the spatial organization of the active site area is the best conserved part of homologous enzymes, while the remaining structure may significantly differ ( *Fig. 1* ) [12-15]. It is widely believed that packing of the polypeptide chain and side chain orientation of the amino acid residues in the active site has a major impact on the ability of an enzyme to recognize, bind, and transform a substrate. Moreover, amino acid residues that impact substrate specificity and catalytic activity generally have been observed within 7-15Å from key catalytic residues [16]. Thus, while studying the relationship between remote homologues it is necessary to perform a bioinformatics analysis in three layers: on the amino acid sequences, three-dimensional structures, and structural organization of the active site areas. A comparative study of the most functionally significant parts of the enzyme structures - the active sites - is of particular interest.

 A computer algorithm has been developed to create a library of enzyme active site structures based on the use of the PDB database, together with numerous programs of structural analysis and identification of functionally important amino acid residues and cavities in an enzyme’s structure. The proposed methodology was used for a comparative bioinformatic analysis of some α,β-hydrolase superfamily members. 

##  METHODS 

**Gathering homologues**

A structure-based similarity to lipase B from *Candida antarctica* search in the PDB databank was performed using the SSM program [8]. Hits were dismissed by the amount of successfully fitted secondary structure elements (at least 30% SSEs should coincide in both the target structure and the query 1TCB). A sequence-based similarity search was performed with the PSI-BLAST program [7] via a nonredundant (nr) sequence dataset. Sequences were clustered at a 95% similarity threshold, and only one representative sequence from a cluster was retrieved.

**Multiple alignment**

Multiple sequence alignment of both the full-size structures and active site areas of enzymes was performed using the t-coffee [17] and Mustang [18] algorithms.

**Visualization**

ThePymol [19] program was used for structural analysis. The Jalview program [20] was used for the representation of primary structure alignments.

**Multiple alignment statistical analysis**

To assess the conservation score of a column *I* in a multiple alignment, the Valdar&Thornton formulation was used: 
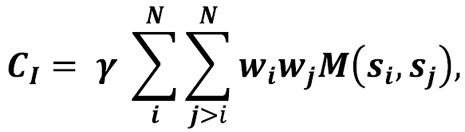
 where M is the amino acids substitution matrix; *s _i_* and *s _j_* – the amino acids in the sequences *i* and *j* of column *I* ; and the coefficient γ is calculated as 
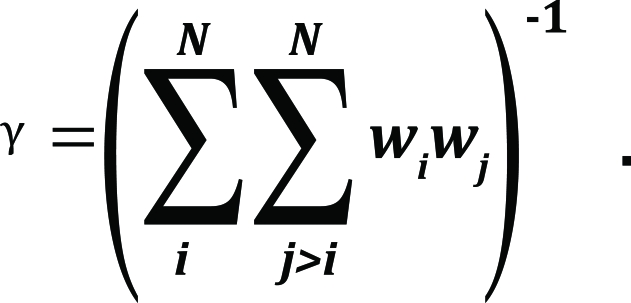


The parameters *w _i_* and *w _j_* refer to the weights of the sequences *i* and *j* as in the Vingron&Argos formulation[22]: 
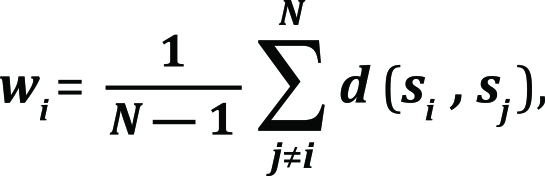
 where *d* is the “genetic” distance between the sequences *i* and *j* calculated in terms of pairwise identities.

 Finally, a Z-score of standard normal distribution was taken as a measure of a column’s conservation: 
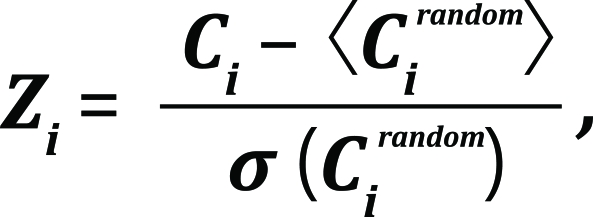
 where *С _I_^rnd^* is the conservation score of a randomly assigned column.

**Fig. 2:**
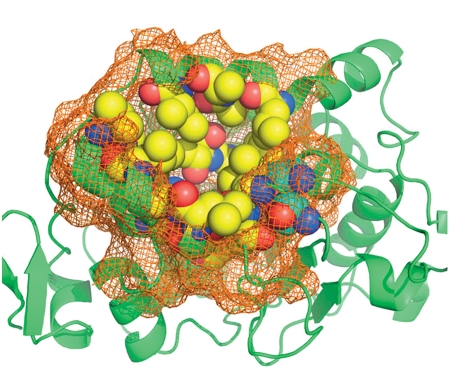
Active site area – a substructure of an enzyme consisting of the amino acid residues involved in substrate delivery, binding, and orientation (yellow), as well as the amino acid residues of the catalytic machinery (blue) and some surrounding residues selected to benefit the integrity of the fragment (showed as dashes).

A Bernoulli rank-order statistics (B-cutoff) was implemented [23, 24] to estimate the statistical significance of the acquired Z-scores. Previously obtained *Z _i_* scores are ordered in decreasing order, and then a rank *k* is computed so that the first *k* scores comprise a set of hits that are the least probable to be observed by chance: 
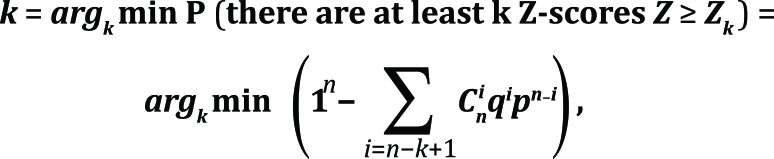
 where *n* is the total number of computed Z-scores, *C _n_ i* is the binomial coefficient, and 
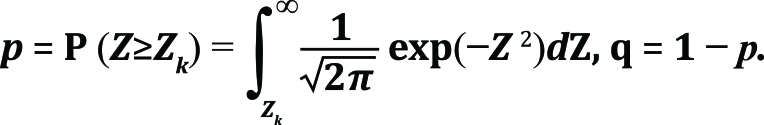


##  RESULTS AND DISCUSSION 

A comparative analysis of the catalytic site organization, as opposed to a full-size structural comparison, could become a source of new crucial information concerning an enzyme’s structure-function relationship. Here, the term “active site” refers to the amino acid residues involved in the catalysis, together with those forming the active site cavity and thus indirectly involved in the catalytic mechanism by interacting with the substrate or “catalytic” amino acid residues. To perform a comparison of enzymes’ active sites, a library of corresponding structures should first be created. A computer algorithm is being proposed to localize and isolate the structure of an enzyme’s active site ( *Fig. 2* ). It consists of three steps:

**1. Identification of the active site residues involved in the catalytic mechanism.**

Amino acid residues are defined as catalytic if they meet any of the following criteria – direct involvement in the catalytic mechanism (for example, as a nucleophile), alteration of the acid-base properties of an active site residue or water molecule directly involved in the catalytic mechanism, and stabilization of the transition state or intermediate of an enzymatic reaction. The Catalytic Site Atlas database (CSA) [25] provides an annotation of the catalytic residues of the enzymes present in the PDB databank. CSA is available to the public at http://www.ebi.ac.uk/thornton-srv/databases/CSA and contains two types of annotated sites: an original hand-annotated set based on information gathered from the literature and an additional homologous set with transferred annotations produced by the PSI-BLAST program [7]. If an enzyme is not listed in the CSA, then catalytically important residues should be gathered manually in the literature or identified using different bioinformatics approaches [26-28].

**2. Identification of the amino acid residues responsible for substrate delivery, binding and orientation in the active site.**

Substrate binding, as a rule, takes place in the so-called structural pockets and cavities on the protein’s surface. Various amino acid residues forming the active site area are not involved directly into the catalytic machinery but interact with the substrate’s functional groups, while diffusion and orientation ensure a productive binding and reactive conformation of the enzyme-substrate complex. The CASTp structural analysis algorithm [29] can be used to complete this step.

**3. Finalizing enzyme active site structural data and the PDB coordinate file.**

 Catalytic residues (determined in step 1) and amino acid residues forming the substrate binding site (determined in step 2) are joined together with surrounding residues, forming secondary-structure elements and intermediate loops. 

 Finally, a substructure of an enzyme is created containing the amino acid residues involved in substrate binding, together with the catalytic amino acid residues and some surrounding residues selected to benefit the integrity of the structural fragment. Technically, it is dumped into the hard drive as a PDB coordinate file with the possibility of including additional information from other databases concerning enzyme structure, function or the peculiarities of its catalytic mechanism. The suggested algorithm could be used to create a library of the active site structures of all enzymes included in the PDB databank. 

**Table 333:** Conserved amino acid residues in active site of lipase B from *Candida antarctica* , serine carboxypeptidase from *Triticum aestivum,* as well as hydroxynitrile lyase from *Hevea brasiliensis * and their homologues

Rank	Z-score	p-value	Position	Alignment column content
1	5.909034	1.496923E-07	224H	HHHHH HHHHH ... HHHHH HH
2	5.909034	1.107511E-14	187D	DDDDD DDDDD ... DDDDD DD
3	5.909034	5.399159E-22	105S	SSSSS SSSSS ... SSSSS SS
4	5.585937	4.061221E-26	39G	GGGGG GGGGG ... GNTTG GG
5	4.976042	1.329205E-25	108G	GGGGG GGGGG ... AAAAA GG
6	3.643481	2.960176E-15	103T	GGGGG GGTTS ... STTSS AG
7	3.077561	7.318560E-12	107G	AAAAA AAGGG ... GGGGG GG
8	2.282191	6.757472E-06	106Q	YYYYY YYQLQ ... YFFYY FF
9	2.097392	2.845755E-05	190V	CCCCC CCCCC ... VVVVL LL
10	1.970983	5.325320E-05	184S	GGGGG GGGGG ... SNNSS NN
11	1.833495	1.540646E-04	80T	AAAAA AAAAA ... GAAVA YY
12	1.525180	8.996767E-03	42T	GGGGG GGGGG ... TRVAG GG
13	1.238283	1.410807E-01	132A	NNNNN NNNNN ... AAAAD DD
14	1.203052	1.191297E-01	133P	GGGGG GGGGG ... PPPPP PG
15	1.173696	9.573976E-02	82Y	DDDDD NNDSN ... QEEQQ YY

Results of bioinformatic analysis are presented in decreasing order of their statistical significance (Z-score). The P-value for a position rank *i * refers to the probability of a result from 1 to *i* to occur in a random sample. Reference position numbering as in 1TCB lipase. Statistical significance threshold is shown in red.

**Fig. 3:**
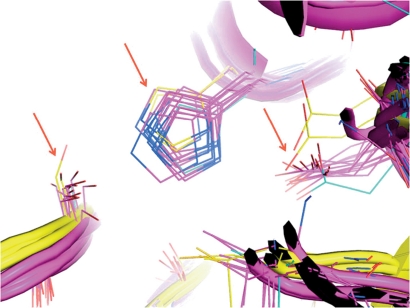
Structural alignment of the active sites of α,β-hydrolase family enzymes: lipase B from *Candida antarctica* , serine carboxypeptidase from *Triticum aestivum* and hydroxynitrile lyase from *Hevea brasiliensis* and their homologues. Conserved residues of the catalytic triad and surrounding loops are indicated with red arrows.

**Fig. 4:**
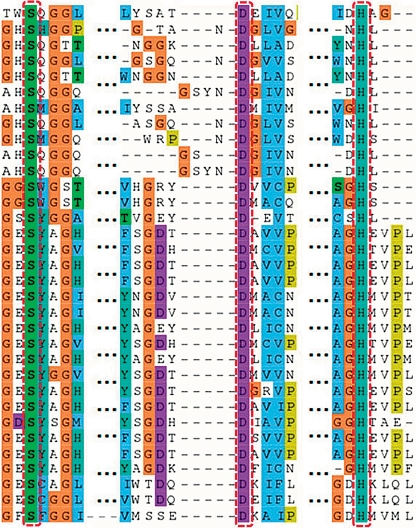
Structural alignment (textual representation) of the active sites of α,β-hydrolase family enzymes: lipase B from *Candida antarctica* , serine carboxypeptidase from *Triticum aestivum* and hydroxynitrile lyase from *Hevea brasiliensis* and their homologues. The sequence is less conserved throughout evolution, compared to structures. Conserved residues of the catalytic triad are indicated with red dashes.

The proposed methodology has been used for a comparative bioinformatic analysis of some α,β-hydrolase superfamily enzymes – lipase B from *Candida antarctica * (PDB code 1TCB) [12], serine carboxypeptidase from *Triticum aestivum * (1WHS) [30], and hydroxynitrile lyase from *Hevea brasiliensis * (1YB6) [13], as well as their homologues established via a combination of iterative sequence searches and structural comparisons (see Methods). The pairwise sequence identity between 1TCB and 1WHS is 7.8%; 1TCB and 1YB6 – 12.4%; and 1WHS and 1YB6 – 13.7%. Such a low sequence identity does not allow to compare distant homologues by sequence alignment. A 3D-Comparison also failed to reveal a significant similarity in the spatial organization of enzyme structures. For example, only catalytic triad residues can be aligned using the SMM program [8], while oxyanion hole residues remain unattended. Oppositely, the Mustang [18] program can align the oxyanion hole residues with catalytic serines but cannot fit other residues of the catalytic triad: histidines and aspartates. With this type of interposition, it remains hard to identify hidden and functionally important regions, while partial manual correction of the alignment does not seem to be a reliable means to improve its quality. The obvious discrepancies in the results obtained using various programs of structural alignment are due to the major differences between the full-size structures of enzymes catalyzing different reactions – only 161 out of 408 amino acid residues of the 1WHS serine carboxypeptidase structure could potentially fit the structure of 1TCB lipase from *Candida antarctica* and 1YB6 hydroxynitrile lyase from *Hevea brasiliensis* . Thus, the proposed procedure was used to prepare the corresponding active site structures for a comparative structural analysis of enzymes so distinct. The resulting files consisted of 170 amino acid residues for 1TCB (54% of the full-size structure), 287 for 1WHS (70%), and 159 for 1YB6 (62%). The analysis of the multiple structural alignment of enzyme active sites revealed a packing similarity between the polypeptide chains, while the organization of the catalytic triad residues was the best conserved – Ser105, His224 and Asp187 (as in 1TCB, see *Table* ). Those positions not only contain the same type of amino acid residues amongst homologues, but they also have similar orientation in the structure ( *Figs. 3, 4* ). Moreover, a geometric comparison of the active sites of the enzymes that catalyze quite diverse chemical reactions revealed a similarity in the organization of the oxyanion hole residues and accompanying loops – part of the structure containing amino acid residue Thr40 in lipase B fits Ile12 in hydroxynitrile lyase and Gly53 in carboxypeptidase. Another oxyanion hole residue – Gln106 in lipase B – that follows the catalytic Ser105 also fits into homologous positions in other enzymes: Tyr147 in carboxypeptidase and Cys81 in hydroxynitrile lyase. The variability of amino acid types in those positions could be justified by taking into account the fact that the NH-group of the main chain peptide bond formed by these residues is involved in the stabilization of the tetrahedral intermediate [12, 13]. The observed structure conservation is especially interesting for hydroxynitrile lyases, since their catalytic mechanism does not involve the formation of a tetrahedral intermediate and its stabilization [31]. Thus, a comparative analysis has helped outline the structural conservation of functionally important active site areas for the evolutionarily remote homologues of α,β-hydrolase superfamily enzymes: lipase B from *Candida antarctica* , serine carboxypeptidase from *Triticum aestivum,* and hydroxynitrile lyase from *Hevea brasiliensis* .

##  CONCLUSIONS 

A computer algorithm has been developed to create a library of enzyme active site structures based on the use of the PDB database, in combination with numerous programs for the structural analysis and identification of functionally important amino acid residues and cavities. The proposed methodology has been used for a comparative bioinformatic analysis of some α,β-hydrolase superfamily enzymes. The comparative analysis helped pinpoint a high similarity in the active site structures of evolutionarily remote homologues of α,β-hydrolase superfamily members – lipase B from *Candida antarctica* , serine carboxypeptidase from *Triticum aestivum* and hydroxynitrile lyase from *Hevea brasiliensis* – despite the low sequence and full-structure identity of these enzymes. A common structural organization of catalytic residues and oxyanion holes was observed between serine carboxypeptidase, lipase B, and hydroxynytrile lyase, despite a significant difference in their functional properties and ability to catalyze diverse chemical transformations. These results demonstrate that a bioinformatic analysis of enzymes and the study of the general principles of biocatalysis should not be limited to sequence and full-structure alignments only. A comparative bioinformatic analysis of the most functionally significant parts of enzyme structures – their active sites – can help uncover resemblances even among remote homologues. This methodology can be used to study the structural organization of the catalytic and substrate-binding sites of various enzymes, as well as to create a database of enzyme active site structures. In addition, the proposed algorithm can be applied when comparing unrelated enzymes with no sequential or structural similarity but with an analogous function developed independently in the course of convergent evolution.
